# Dissection of the Temporofrontal Extreme Capsule Fasciculus Using Diffusion MRI Tractography and Association with Lexical Retrieval

**DOI:** 10.1523/ENEURO.0363-23.2023

**Published:** 2024-01-19

**Authors:** E. B. Barbeau, A. Badhwar, S. Kousaie, P. Bellec, M. Descoteaux, D. Klein, M. Petrides

**Affiliations:** ^1^Cognitive Neuroscience Unit, Montreal Neurological Institute, McGill University, Montreal, Quebec H3A 2B4, Canada; ^2^Center for Research on Brain, Language and Music (CRBLM), Montreal, Quebec H3G 2A8, Canada; ^3^Département de pharmacologie et physiologie, Faculté de médecine, Université de Montréal, Montreal, Québec H3C 3J7, Canada; ^4^Institut de génie biomédical, Université de Montréal, Montréal, Québec H3C 3J7, Canada; ^5^Centre de Recherche de l’Institut Universitaire de Gériatrie de Montréal (CRIUGM), Montreal, Québec H3C 3J7, Canada; ^6^Département de Psychologie, Université de Montréal, Montréal, Québec H3C 3J7, Canada; ^7^Sherbrooke Connectivity Imaging Lab (SCIL), Computer Science Department, Université de Sherbrooke, Sherbrooke, Quebec J1K 2R1, Canada; ^8^ Departments of Neurology and Neurosurgery; ^9^Psychology, McGill University, Montreal, Quebec H3A 1G1, Canada

**Keywords:** diffusion imaging tractography, language, lexical retrieval, temporofrontal extreme capsule fasciculus, ventral stream, white matter

## Abstract

The well-known arcuate fasciculus that connects the posterior superior temporal region with the language production region in the ventrolateral frontal cortex constitutes the classic peri-Sylvian dorsal stream of language. A second temporofrontal white matter tract connects ventrally the anterior to intermediate lateral temporal cortex with frontal areas via the extreme capsule. This temporofrontal extreme capsule fasciculus (TFexcF) constitutes the ventral stream of language processing. The precise origin, course, and termination of this pathway has been examined in invasive tract tracing studies in macaque monkeys, but there have been no standard protocols for its reconstruction in the human brain using diffusion imaging tractography. Here we provide a protocol for the dissection of the TFexcF in vivo in the human brain using diffusion magnetic resonance imaging (MRI) tractography which provides a solid basis for exploring its functional role. A key finding of the current dissection protocol is the demonstration that the TFexcF is left hemisphere lateralized. Furthermore, using the present dissection protocol, we demonstrate that the TFexcF is related to lexical retrieval scores measured with the category fluency test, in contrast to the classical arcuate fasciculus (the dorsal language pathway) that was also dissected and was related to sentence repetition.

## Significance Statement

In addition to the arcuate fasciculus that constitutes the classic dorsal stream for language, there has been recent interest in a ventral network of language processing. The white matter tract forming this ventral stream is the temporofrontal extreme capsule fasciculus (TFexcF), but in MRI studies it is frequently mixed with other adjacent white matter tracts making it difficult to examine its functional role. First, we aim to provide a detailed and anatomically accurate protocol to dissect the TFexcF in vivo using diffusion MRI tractography. Second, in the 50 participants in whom we dissected the TFexcF, we explored correlations with behavioral measures. We show that the TFexcF is involved in lexical retrieval, while the arcuate fasciculus relates to overt language production.

## Introduction

Understanding the various cortical regions that constitute the language network has been the focus of numerous studies over the years (Geschwind, 1970; Hickok and Poeppel, 2004; Price, 2012; Friederici et al., 2017). The classical language regions that include Broca's region in the inferior frontal gyrus and Wernicke's area in the posterior superior temporal gyrus (Petrides, 2014) are anatomically connected by the arcuate fasciculus (AF; Petrides and Pandya, 1988, 2006, 2009; Barbeau et al., 2020) and are part of the dorsal network of language, which has been mainly associated with mapping sound to articulation and phonological processing (Hickok and Poeppel, 2004; Saur et al., 2008). More recently, there has been intense interest in a ventral network of language processing (Hickok and Poeppel, 2004; Frey et al., 2008; Saur et al., 2008; Brauer et al., 2013; Lopez-Barroso and de Diego-Balaguer, 2017; Ries et al., 2019) connecting lateral mid-temporal areas with the lateral frontal lobe, and this network has been discussed in relation to mapping sound to meaning, as well as with selective verbal retrieval (Klein et al., 1995, 1997; Petrides et al., 1995; Saur et al., 2008; Petrides, 2015, 2023). The anatomical pathway of the ventral stream of language is the temporofrontal extreme capsule fasciculus (TFexcF) which is a *monosynaptic* fasciculus linking mid-lateral temporal cortex with lateral frontal cortex via axons that course through the extreme capsule. The lack of a standard dissection protocol to reconstruct the TFexcF in the human brain has been a major impediment to examination of its role in language. Accurate dissection of the distinct monosynaptic tracts is essential for research aimed at examining their functional contributions and at investigating pathological conditions that affect these pathways.

Although recent advances in image processing algorithms for diffusion tractography in human brains permit improved modeling of crossing fibers, it is important to note that, just as with classical white matter dissections (Agrawal et al., 2011; Arnts et al., 2014), current diffusion magnetic resonance imaging (MRI) tractography methods cannot establish the precise cortical origins and terminations of particular white matter tracts, and other limitations have to be considered (Maier-Hein et al., 2017). Technical limitations of MRI tractography methods also make it impossible to determine whether connections from one brain area to another are monosynaptic via a specific fasciculus or comprise a polysynaptic series of connections (see Campbell and Pike, 2014 for a review). Given these limitations, the use of invasive anatomical tracers placed in specific cortical areas of the macaque monkey remains the gold standard for examining precise anatomical connectivity between regions of the primate cerebral cortex (Petrides and Pandya, 1984, 1988, 2009). Although macaques do not possess language in the human sense, the cytoarchitectonic homologs of the areas that in the language-dominant hemisphere of the human brain become important for specific aspects of language processing have been demonstrated in the macaque brain (Petrides and Pandya, 2009; Petrides, 2014). Thus, the precise connectivity established in macaque monkey studies provide strong hypotheses to be examined in the human brain using diffusion MRI tractography.

The TFexcF was discovered in an experimental anatomical tracing study in the macaque monkey that demonstrated that the anterior to intermediate part of the lateral temporal cortex is connected with various ventrolateral and dorsolateral frontal cortical areas via axons that course in the extreme capsule (Petrides and Pandya, 1988, 2002a,b). The present study aims to provide the scientific community with an anatomically accurate protocol for the reconstruction of the TFexcF from the anterior to intermediate temporal areas that give rise to this monosynaptic fasciculus. Furthermore, the present study examines the functional relation of the left TFexcF in lexical retrieval, which has been predicted to be a major contribution of the ventral stream of language (Petrides, 2014), by correlating the structural properties of this tract with behavioral measures of language performance.

## Material and Methods

### Participants

The study was based primarily on an in-house dataset of 50 right-handed healthy volunteers (mean age, 23.9 years; range, 18–34 years; 24 females). All participants scored within the normal range of intellectual functioning on the Matrix Reasoning subtest of the Wechsler Adult Intelligence Scale (group mean score, 19.4; range, 9–25). Participants were recruited from the Montreal area and were included if they reported a high degree of current usage and proficiency in English. The exclusion criteria were any neurological, psychiatric, or other medical condition and medication known to affect brain structure or function. All methods were carried out in accordance with the relevant guidelines and regulations. Written informed consent was obtained from all participants, and all experimental protocols were performed according to the Declaration of Helsinki and approved by the Research Ethics Board of the Montreal Neurological Institute (MNI). A financial compensation was offered to the participants.

### Validating in an independent dataset

In order to ensure that the present protocol can be replicated in different datasets with different diffusion weighted image acquisition parameters, we also dissected the TFexcF in eight participants randomly selected from the Human Connectome Project (HCP) dataset (www.humanconnectome.org) in a subsample of healthy right-handed participants within the same age range as our in-house dataset.

### Data acquisition

#### In-house dataset

MRI data were acquired from all 50 participants at the McConnell Brain Imaging Center of the MNI on a Siemens 3 T Tim Trio Scanner using a 32-channel head coil. The scanning session included the acquisition of diffusion-weighted MRI data (73 slices; TR = 10,000 ms; TE = 90 ms; 2 mm^3^ voxels; slice thickness of 2 mm; directions = 64; *b* = 1,000 s/mm^2^), as well as a T1-weighted structural scan obtained with an MPRAGE sequence (192 slices; TR = 2,300 ms; TE = 2.98 ms; flip angle = 9°).

#### HCP dataset

The HCP dataset was acquired on multiple Siemens platforms. The diffusion-weighted MRI scans had the following scanning parameters: 90 diffusion-encoding directions (*b* values of 1,000, 2,000, 3,000 s/mm^2^) plus 6 *b* = 0 acquisitions; voxel size, 1.25 × 1.25 × 1.25 mm; slices, 111; TR, 5,520 ms; and TE, 89.5 ms (www.humanconnectome.org). Structural scanning included a T1-weighted MPRAGE with voxel size, 0.7 × 0.7 × 0.7 mm^3^; TR, 2,400 ms; TE, 2.14 ms; TI, 1,000 ms; and 8° flip angle.

### Diffusion-weighted image analyses

#### In-house dataset

Reconstruction of fiber orientation distribution functions (ODFs; Descoteaux et al., 2009) was carried out using Dipy (Garyfallidis et al., 2014) followed by anatomically constrained probabilistic tractography (Girard et al., 2014), seeding from the white matter and white matter/gray matter interface (10 seeds per voxel). All streamlines looping onto themselves at 300 degrees or more were removed. For details on diffusion data processing, see Barbeau et al. (2020) in the section on Probabilistic HARDI tractography.

#### HCP dataset

Diffusion MRI and structural MRI (T1) data were submitted to the diffusion tractography pipeline described in Girard et al. (2014). The diffusion MRI data were upsampled to the T1 isotropic resolution using spline interpolation (Dyrby et al., 2014). Standard DTI metrics and fiber orientation density functions of spherical harmonics order 8 were reconstructed with Dipy (Garyfallidis et al., 2014). T1 data were nonlinearly warped into upsampled diffusion space using ANTs (Avants et al., 2008). White matter (wm), gray matter (gm), and CSF partial volume estimations were obtained using FSL FAST (Zhang et al., 2001) and the wm/gm interface and tracking domain defined for probabilistic tracking using PFT (Girard et al., 2014) and 10 seeds/voxel of the interface. The full brain tractogram, for dissection purposes, was nonlinearly warped to the MNI stereotaxic space by applying the ANTs transformation to the streamline points.

### Diffusion MRI tractography—tract reconstructions

TrackVis (Ruopeng Wang, Van J. Wedeen, TrackVis.org, Martinos Center for Biomedical Imaging, Massachusetts General Hospital) was used for region of interest (ROI) placement and tract dissections.

#### The temporofrontal extreme capsule fasciculus

**Figure 1.**
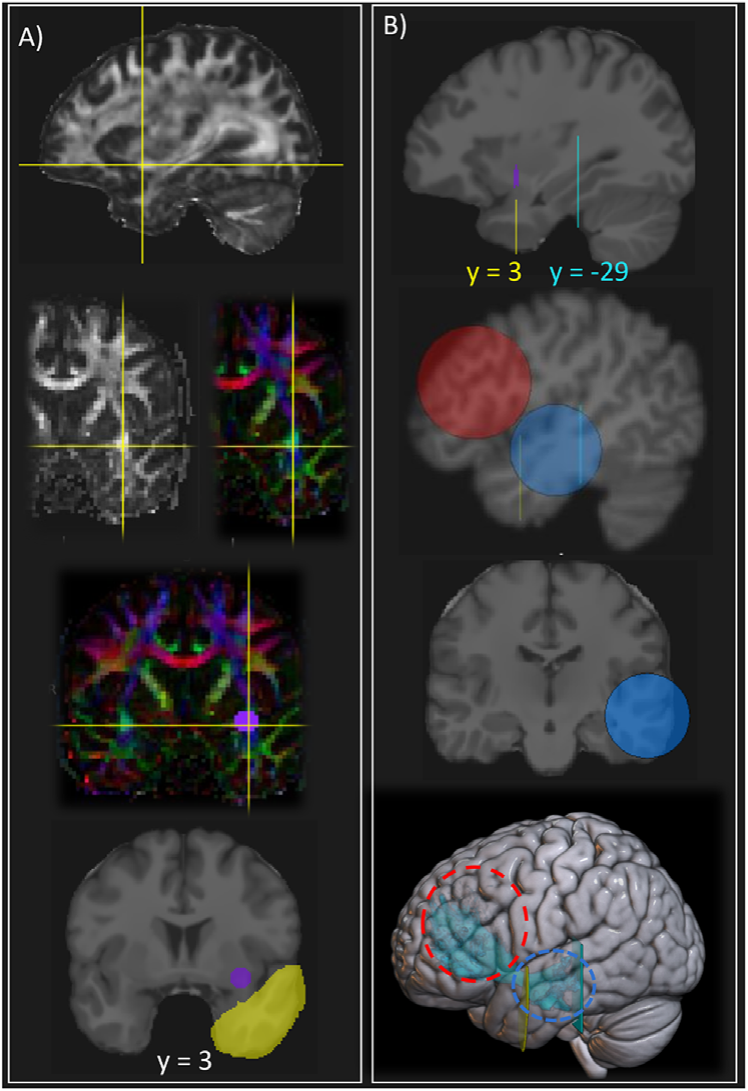
Inclusion and exclusion ROIs used to dissect the temporo-frontal extreme capsule fasciculus (TFexcF). ***A***, Location of the extreme capsule on the FA and FA and RGB-color map and placement of the extreme capsule inclusion ROI (purple). ***B***, Placement of the frontal (red) and temporal (blue) inclusion spheres and position of the exclusion ROIs for the anterior temporal (yellow) and posterior temporal (cyan) regions.

First, on the FA or RGB (fractional anisotropy, red–green–blue) color map, the extreme capsule was localized on the coronal plane by finding the brightest point, situated immediately posterior to the point where the temporal and frontal lobes start to join (Fig. 1*A*). A circular plane ROI was drawn to encompass the bright region (Fig. 1, purple ROI, around MNI *y* = 3). Because of the point-based streamline selection methods in TrackVis (Rheault et al., 2017), we drew the ROI on four adjacent coronal slices to ensure the selection of all streamlines passing within “any-part” of the ROI.

**Figure 2.**
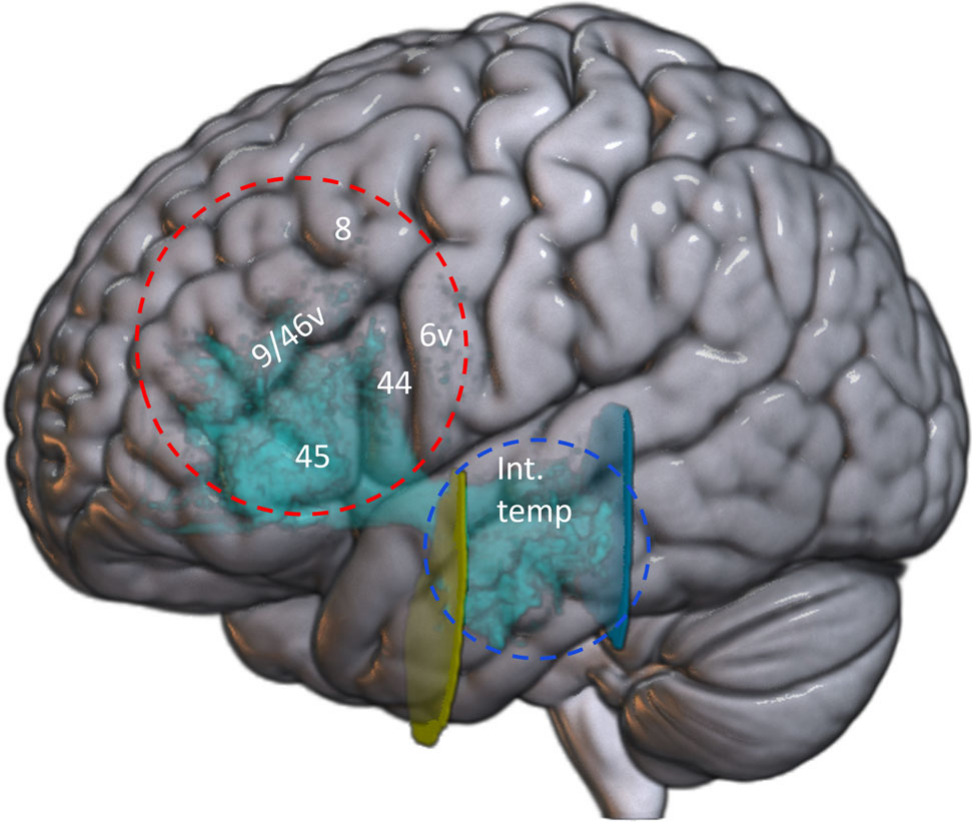
Representation on the 3D MNI brain of the cortical areas encompassed by the frontal (red dotted circle) and temporal (blue dotted circle) inclusions ROIs for the dissection of the TFexcF and the limits (yellow and cyan vertical planes) of the TFexcF intermediate temporal (int. temp) origins and terminations.

The frontal inclusion ROI was a 30 mm sphere (radius centered at MNI coordinates [*x* = −53, *y* = 27, *z* = 20] for the left hemisphere and at [*x* = 49, *y* = 27, *z* = 20] for the right hemisphere) using the “Either end” option (Fig. 1*B*, red sphere). This ROI is designed to include the fibers connecting the pars opercularis (area 44) and pars triangularis (area 45) of the inferior frontal gyrus (IFG) and the adjacent ventral premotor cortex (ventral area 6) and the region of the middle frontal gyrus (MFG) where areas 8A and 9/46 lie. The temporal ROI was designed to include the fibers ending in the intermediate part of the temporal lobe only (excluding the temporal pole, which is connected with the orbitofrontal region via the uncinate fasciculus, and the posterior temporal areas which are connected with the lateral frontal areas by the arcuate fasciculus; see Fig. 2 for a schematic representation of the cortical areas encompassed by the sphere ROIs). This 25 mm sphere was centered at MNI coordinates [*x* = −57, *y* = −15, *z* = −12] for the left hemisphere and [*x* = 56, *y* = −17, *z* = −10] for the right hemisphere (Fig. 1*B*, blue ROI). To ensure that the streamlines were restricted to the intermediate temporal areas only, we added an exclusion ROI (Fig. 1*B*, cyan ROI) in the coronal view, immediately posterior to the last slice where Heschl's gyrus was visible, around MNI *y* = −29. Note that this ROI is the same one used for the arcuate fasciculus reconstruction to exclude fibers from more anterior parts of the temporal lobe. To exclude the fibers belonging to the uncinate fasciculus (originating from the temporopolar region), a plane exclusion ROI was used at the level of the temporal lobe in the same coronal plane below the extreme capsule (Fig. 1*A* and *B*, yellow ROI, around MNI *y* = 3). For all exclusion ROIs, the TrackVis condition “No part” was chosen. Occasionally, additional exclusion ROIs were used on a case-by-case basis for aberrant streamlines clearly not belonging to the tract. The final result hiding the two inclusion spheres is displayed for one participant in Figure 1. The same procedure was used to dissect the tracts in the left and right hemispheres.

#### Validating with the arcuate fasciculus

In order to dissociate the structure–function relationships of the TFexcF and the AF (i.e., the two temporofrontal language processing fasciculi), we examined the properties of the left AF in the same participants. The arcuate fasciculus which forms the dorsal stream of language and connects the posterior temporal areas with the inferior frontal areas was reconstructed according to the protocol described in detail in Barbeau et al. (2020). In the frontal lobe, an end-point mask was manually drawn around the inferior frontal gyrus to include the white matter connecting areas 44, 45, and the rostral part of ventral area 6. The temporal end-point ROI was specifically placed to restrict the fibers to the posterior part of the temporal lobe region that lies caudal to Heschl's gyrus. Only fibers ending in both the frontal and temporal ROIs and passing within the temporoparietal white matter and curving around the Sylvian fissure were included.

### Behavioral measures

The language measures included tests of sentence repetition, letter fluency, and category fluency. The Recalling Sentences subtest of the Clinical Evaluation of Language Fundamentals – fourth edition (CELF-4; Semel et al., 2003) was used to measure sentence repetition. Participants had to repeat sentences immediately after they were read out loud by the experimenter. Each sentence was scored out of 3, with 3 representing no errors and 0 representing four or more errors in the repetition of the sentence. Letter fluency was tested by asking participants to produce as many English words as they could think of in 1 min, starting with a specific letter of the alphabet (*F*, *A*, and *S*). Participants were instructed that proper nouns, numbers, or words differing from an accepted word only in terms of suffix (e.g., surf, surfer, surfing) would be excluded. The total number of words produced was used as the final score. In the category fluency task, participants were asked to produce as many English words/exemplars as possible from a specific category, that is, animals, in 1 min. Each participant's score reflects the total number of correct animal names produced.

### Statistical analyses

Paired-sample *t* tests were performed to compare the properties of each tract in the left and in the right hemispheres in order to investigate whether the TFexcF was left-lateralized as might be expected for a white matter tract involved in language in right-handed individuals.

Pearson correlation coefficients were computed between the properties of the white matter tracts (left TFexcF and arcuate fasciculus) and behavioral language measures (category fluency, letter fluency, and sentence repetition) in order to explore whether there were differences between the tracts in their association with particular aspects of language performance.

## Results

### TFexcF reconstruction

#### In-house dataset

**Figure 3.**
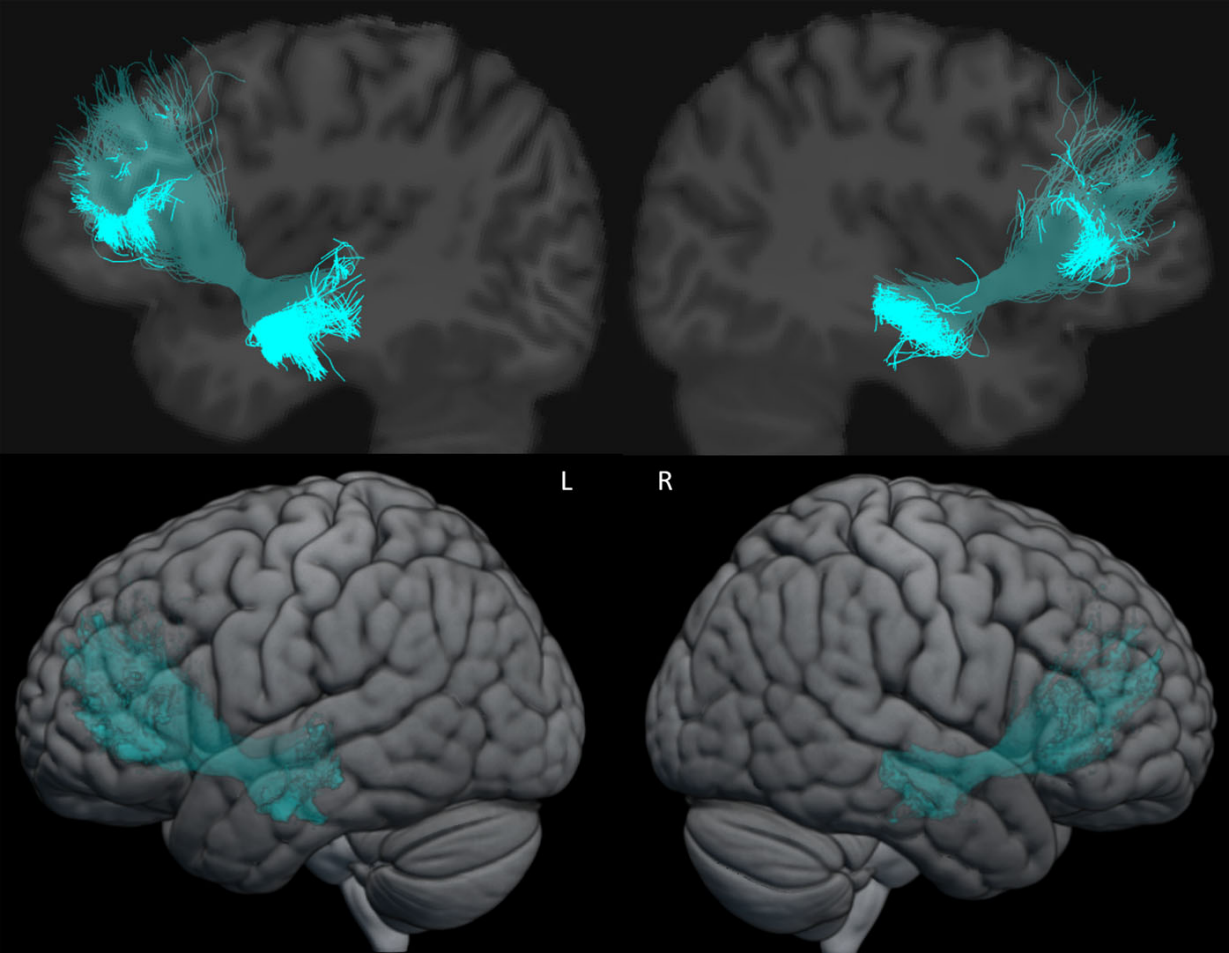
Example of the dissection of the left and right TFexcF in one participant overlayed on the participant's T1 image normalized to the MNI-152 template (top) and on the MNI-152 3D template (bottom). L, left hemisphere; R, right hemisphere.

**Figure 4.**
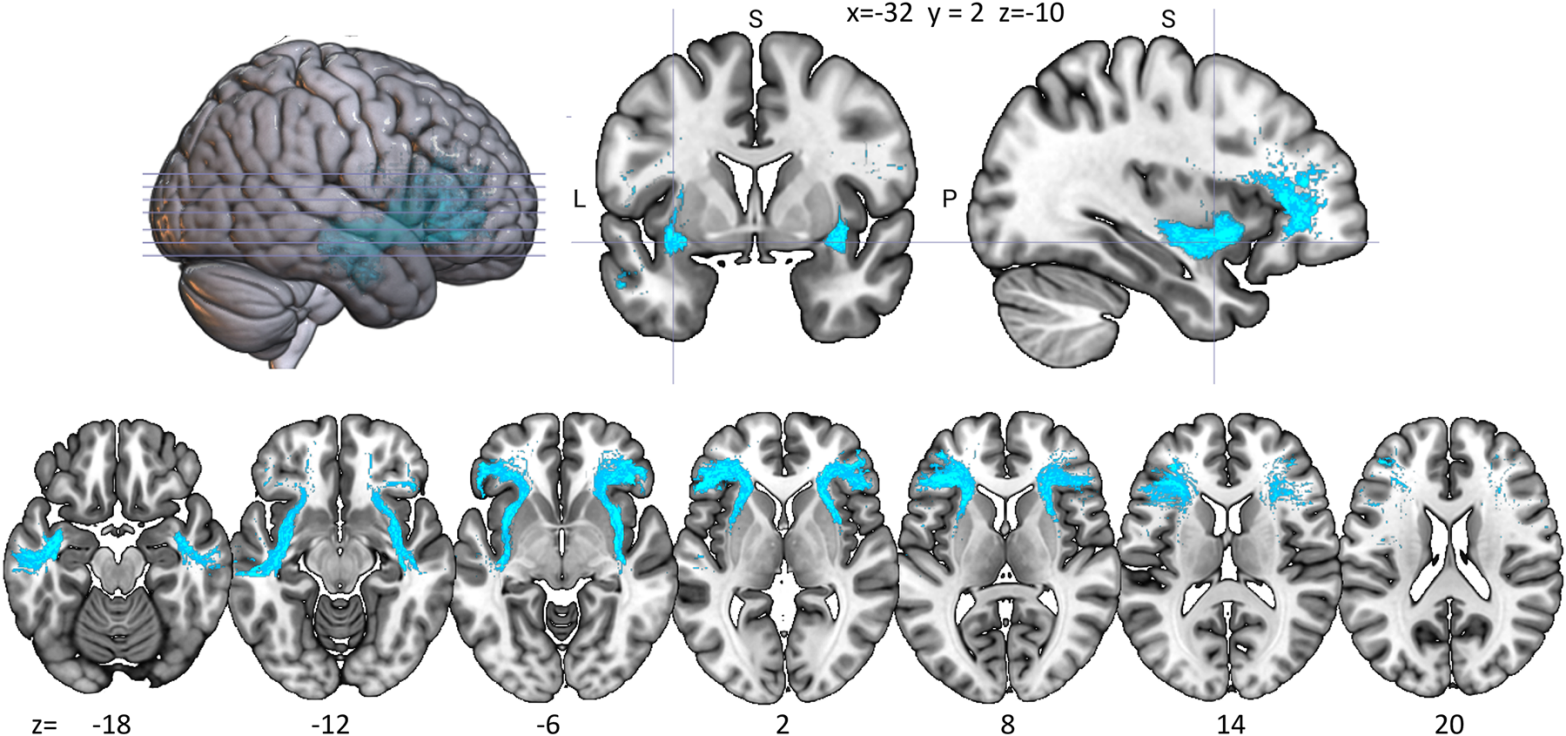
The left and right TFexcF of 12 participants overlayed on the MNI-152 brain template. Lighter color indicates spread of overlap. See Figure 4-1. For left and right TFexcF in eight participants from the HCP dataset overlayed on the MNI brain template.

The left and right TFexcF were dissected in all 50 participants. Figure 3 displays an example of the dissected left and right TFexcF in one participant. For visualization purposes, Figure 4 shows in 12 randomly selected participants, their tracts overlaid on the average MNI brain. Figure 4 displays the left and right TFexcF for those participants, showing the trajectory of the tract from intermediate temporal areas, going through the extreme capsule underneath the anterior insula and ending within lateral frontal areas.

10.1523/ENEURO.0363-23.2023.f1-1 Figure 4-1 Left and right temporo-frontal extreme capsule fasciculus in 8 participants from the HCP dataset overlayed on the MNI brain template. Download Figure 4-1, TIF file.

#### Validation (HCP) dataset

To assess generalizability of the method, in addition to our own dataset, we dissected the TFexcF in the left and right hemispheres in eight randomly selected participants from a separate dataset (the HCP dataset; www.humanconnectome.org). See Figure 4-1 showing the resulting left and right hemisphere TFexcF of all eight participants overlaid on the MNI template. The present protocol can thus be applied to other datasets with differing acquisition parameters.

### TFexcF tract properties—in-house dataset

Tract properties, including overall volume (range *L*, 811–14,831 mm^3^; *R*, 363–13,388 mm^3^), FA (range *L*, 0.338–0.607; *R*, 0.334–0.579), mean diffusivity (MD, range *L*, 6.9 × 10^−4^–7.9 × 10^−4^; *R*, 6.8 × 10^−4^–8.3 × 10^−4^ mm^2^/s), axial diffusivity (AD, range *L*, 1.00 × 10^−3^–1.29 × 10^−1^; *R*, 1.06 × 10^−3^–1.29 × 10^−3^ mm^2^/s), and radial diffusivity (RD, range *L*, 4.2 × 10^−4^–6.3 × 10^−4^; *R*, 4.3 × 10^−4^–6.6 × 10^−4^ mm^2^/s), were extracted for each participant from the in-house dataset, with mean values displayed in Table 1. There were no significant differences in any of the measures between male and female participants (independent-sample tests: all *p* values between 0.074 and 0.899).

**Table 1.** Mean (*M*) and standard deviation (SD) values (in the 50 participants) for the left (*L*) and right (*R*) TFexcF

TFexcF		*L*	*R*
Volume	*M*	5,670.96	3,732.48
	*SD*	3,754.10	2,718.00
FA	*M*	0.517	0.503
	*SD*	0.057	0.057
MD	*M*	7.35 × 10^−4^	7.42 × 10^−4^
	*SD*	2.34 × 10^−5^	2.90 × 10^−5^
AD	*M*	1.20 × 10^−3^	1.20 × 10^−3^
	*SD*	5.76 × 10^−5^	4.67 × 10^−5^
RD	*M*	5.02 × 10^−4^	5.12 × 10^−4^
	SD	4.55 × 10^−5^	5.05 × 10^−5^

Volume (mm^3^); FA, fractional anisotropy; MD, mean diffusivity (mm^2^/s); AD, axial diffusivity (mm^2^/s); RD, radial diffusivity (mm^2^/s).

### Lateralization—in-house dataset

#### Paired sample *t* tests

**Figure 5.**
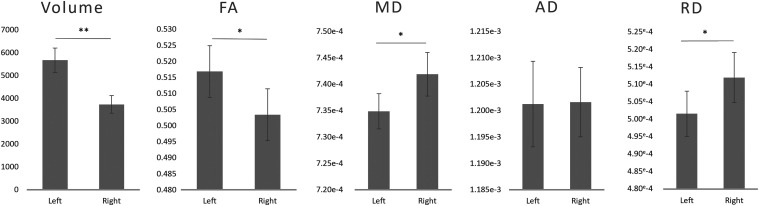
Mean left and right TFexcF properties. Volume (mm^3^); FA, fractional anisotropy; MD, mean diffusivity (mm^2^/s); AD, axial diffusivity (mm^2^/s); RD, radial diffusivity (mm^2^/s). Error bars represent the standard error of the mean. *p < 0.05 and **p < 0.001 for left–right differences from independent-sample *t* tests.

The volume (*t *= 4.789; *p *< 0.001) and FA (*t *= 2.937; *p *= 0.005) values were significantly larger in the left than those in the right hemisphere, while MD (*t *= −2.55; *p *= 0.014) and RD (*t *= −2.49; *p *= 0.016) values were larger in the right hemisphere. AD values did not differ across hemispheres. See Figure 5 for results.

### Correlations with behavioral measures—in-house dataset

The mean scores for all 50 participants were 22.34 (*SD* = 6.4) for category fluency, 39.42 (*SD* = 12.4) for letter fluency, and 59.88 (*SD* = 9.0) for sentence repetition (information for sentence repetition scores was missing for two participants).

The properties (FA and RD) of the left TFexcF were significantly correlated with category fluency scores (Table 2) but not with the letter fluency or sentence repetition scores.

**Table 2.** Pearson's correlation values (*r*) and *p* values between the behavioral language measures and the properties of the left TFexcF and the left ventral branch of the arcuate fasciculus (AFv)

			Category fluency	Letter fluency	Sentence repetition
TFexcF	FA	*r*	**0.349** *	0.066	0.260
		*p*	**0.013**	0.646	0.074
	MD	*r*	−0.090	−0.009	−0.029
		*p*	0.532	0.949	0.842
	AD	*r*	**0.351** *	0.053	**0.285** *
		*p*	**0.012**	0.715	**0.049**
	RD	*r*	**−0.292** *	−0.041	−0.201
		*p*	**0.040**	0.780	0.167
AFv	FA	*r*	0.224	0.060	**0.334** *
		*p*	0.117	0.679	**0.020**
	MD	*r*	0.082	0.082	−0.033
		*p*	0.573	0.573	0.823
	AD	*r*	**0.318** *	0.061	**0.372** **
		*p*	**0.024**	0.671	**0.009**
	RD	*r*	−0.107	−0.004	−0.242
		*p*	0.458	0.977	0.097

FA, fractional anisotropy; MD, mean diffusivity; AD, axial diffusivity; RD, radial diffusivity. Significant correlations are in bold.

* *p *< 0.05.

** *p *< 0.001.

To verify whether this association was specific to the left TFexcF, we also examined correlations with the properties of the left arcuate fasciculus. The FA of the left arcuate fasciculus correlated only with sentence repetition scores. AD also correlated with sentence repetition scores, but this property was not specific to any tract or behavioral measure as the AD of both the TFexcF and arcuate fasciculus correlated with both the category fluency and sentence repetition scores.

## Discussion

The present study provides a protocol for reconstructing the TFexcF, that is, the ventral temporofrontal pathway that has recently become of great interest in relation to a ventral stream of language processing (Hickok and Poeppel, 2004; Saur et al., 2008). The TFexcF is distinct from the classical arcuate fasciculus that connects dorsally the posterior temporal region with Broca's area (Barbeau et al., 2020) via fibers arching around the end of the Sylvian fissure. Both these pathways were reconstructed in the present investigation and related to performance on language tasks to address questions that have been raised regarding the role of the ventral language network (Hickok and Poeppel, 2004). The TFexcF was shown to be left hemisphere lateralized in the sample of right-handed participants examined, providing further support for the importance of this ventral white matter pathway in language processing. Furthermore, it was related to lexical retrieval scores measured with the category fluency test. For comparison with the TFexcF, the classical arcuate fasciculus (the dorsal language pathway) was also reconstructed and was shown to be related to sentence repetition. These findings are consistent with predictions about the role of the ventral network for language in certain aspects of mapping meaning to auditory processing, such as assigning meaning to lexical items, as well as in selective lexical retrieval (Klein et al., 1995, 1997; Petrides et al., 1995; Saur et al., 2008; Petrides, 2015, 2023).

The TFexcF had originally been demonstrated in macaque gold standard invasive tracer studies. It originates from the intermediate mid-temporal lateral cortical region (excluding the temporal pole) and courses through the extreme capsule (the white matter just below the anterior insula) to target various areas within the lateral frontal cortex via one-step monosynaptic axonal connections (Petrides and Pandya, 1988, 2009). Furthermore, injections of retrograde tracers in the various lateral frontal cortical areas demonstrated the cells of origin of the temporofrontal connections that constitute the TFexcF (Petrides and Pandya, 1999, 2002b). For example, injections within the ventrolateral frontal cortical area 45 (i.e., the cytoarchitectonic homolog of the pars triangularis area 45 in the human brain) demonstrated labeled neurons within the anterior and intermediate lateral temporal cortical areas, but there were no connections with the visual occipital cortical areas (i.e., Brodmann areas 17, 18, 19). The TFexcF axons were completely distinct from those originating in the posterior superior temporal gyrus and which arch around the posterior Sylvian fissure and constitute the classic arcuate fasciculus (Petrides and Pandya, 1988, 2009), as well as those originating from the rostralmost part of the temporal lobe (the temporopolar region) and which are directed to the orbitofrontal cortical areas via the uncinate fasciculus (Petrides and Pandya, 1988, 2009).

In the current study, this monosynaptic TFexcF pathway was reconstructed in the human brain as a tract that is distinct from the inferior fronto-occipital fasciculus (IFOF). Because of the limitations of current diffusion imaging tractography methods, the IFOF is often described as a continuous pathway (Conner et al., 2018), but precise tracer injections in macaque monkey studies have shown that it is a multistep polysynaptic pathway. The occipital primary sensory visual cortical area 17 (V1) is connected with the second visual cortical area 18 (V2), and then there are short connections to functional visual areas located within cytoarchitectonic area 19 and, finally, connections to posterior and then anterior inferior temporal cortex where higher level perceptual processing of visual information is carried out (Tusa and Ungerleider, 1985). Thus, early visual areas within the occipital lobe process visual sensory information that is then relayed via multisynaptic steps to the inferior temporal region (Petrides and Pandya, 2002a) and finally these inferior temporal areas project to the frontal cortex. Clearly, this multistep processing of visual information originating in the occipital lobe and processed in posterior and inferior temporal areas cannot be directly related to language information processing. Regardless of whether the IFOF is a direct or a polysynaptic stream of shorter fasciculi originating within the occipital cortex, the fundamental scientific question for language research must be the reconstruction of the *monosynaptic* TFexcF that originates within the intermediate temporal cortex (i.e., the superior and middle temporal gyri that process language information). Furthermore, this TFexcF must clearly be separated from the uncinate fasciculus that originates from the temporal polar and adjacent ventromedial anterior temporal region that targets orbitofrontal cortical areas. More recently, these pathways have also been reconstructed in the human brain with diffusion MRI (Frey et al., 2008; Saur et al., 2008; Weiller et al., 2021). Frey et al. (2008) examined only the connection from a specific part of the superior temporal gyrus with subject-specific hand-drawn ROIs making it a partial reconstruction of the tract. They also restricted examination of the frontal end-points to areas 44 and 45 (Broca region), but we know from gold standard macaque monkey injections that this pathway targets, in addition, dorsolateral frontal areas (Petrides and Pandya, 1999). Note that the present study examined the connections via the TFexcF from the entire intermediate lateral temporal cortex and the complete termination points in the lateral frontal cortex, including connections to the areas of the middle frontal gyrus which are also connected with the temporal lobe through the TFexcF (Petrides and Pandya, 1988, 2007). Saur et al. (2008) did not specify the anatomical temporal end-points, that is, the end-points were functionally defined from task-specific clusters of brain activation. Here our aim was to provide specific anatomical reconstructions of the complete TFexcF using inclusion ROIs in the MNI standard stereotaxic space for universal replicability and to contribute to further investigation of the functional role of this tract for language. In a previous combined functional MRI and diffusion MRI investigation (Barbeau et al., 2023) of a subsample of the participants from the present study, it was shown that the properties of the reconstructed TFexcF were related to functional activation of frontal area 9/46v and intermediate temporal cortex during the monitoring of information in a working memory task consistent with the anatomical areas it connects.

Very few studies investigated the effect on cognition of specific lesions of the TFexcF, and these were generally related to language deficits associated with aphasias (Martinez Oeckel et al., 2021). In a recent study, Kourtidou et al. (2022) carefully examined language and overall cognitive performance of a patient with chronic middle cerebral artery cerebrovascular accident who had a well-documented lesion restricted to the anterior to intermediate lateral temporal cortex and its connections via the extreme capsule with the pars triangularis of the inferior frontal gyrus. The performance of this unique patient was compared with that of two patients who had chronic cerebrovascular accidents of the middle cerebral artery with damage to the classic dorsal posterior temporoparietal language system. The patient with the anterior temporofrontal lesion via the TFexcF had excellent and fluent speech, but selective impairment in accessing lexico-semantic information. This finding was distinct from the classic impairment in sentence comprehension and speech repetition of patients after lesions in the dorsal language system. Earlier functional neuroimaging studies had shown that the mid-ventrolateral frontal region (area 45) via interaction with the intermediate lateral temporal language regions is critical for the selective retrieval of language-related information (Klein et al., 1995, 1997; Petrides et al., 1995; Kostopoulos and Petrides, 2003, 2008, 2016), and these frontotemporal functional interactions are most likely occurring via the TFexcF. It is of interest that electrical stimulation in the TFexcF can lead to semantic paraphasias, such as the substitution of a target word by a semantically related word (e.g., substitution of cat for dog; Duffau et al., 2005), and Klein and colleagues showed selective disruption of lexical retrieval in a patient undergoing intraoperative cortical stimulation mapping in the inferior frontal region (Klein et al., 1997).

The present results are consistent with previous findings in that performance on the controlled retrieval category fluency task was correlated with the properties of the TFexcF, but not with the properties of the arcuate fasciculus which was, as predicted, related with the sentence repetition scores. This is also in line with the well-documented role of the arcuate fasciculus in mapping sound to articulation (Saur et al., 2008), as well as evidence from lesion case studies in patients demonstrating that damage to the left arcuate fasciculus leads to specific impairments in sentence repetition (Damasio and Damasio, 1980). It has also been suggested that the ventral stream of language may not be involved exclusively with lexical retrieval, but more generally with conceptual processing and categorical classification and categorical decisions (Weiller et al., 2022), in line with the association we found with the category fluency task. The absence of correlation between the properties of the TFexcF and the letter fluency test is not unexpected since letter fluency would rely more on the dorsal phonological system involving supramarginal areas and its link to the frontal lobe through the third branch of the superior longitudinal fasciculus (SLF III) independent of semantic knowledge associated with the words.

In summary, we provide a clear protocol for the dissection of the TFexcF in the human brain using diffusion imaging tractography. The use of inclusion and exclusion ROIs placed in specific locations according to anatomical priors provided by comparative cytoarchitectonic analysis of macaque monkey with human cortical areas, as well as gold standard corticocortical connectivity studies in the macaque (Petrides and Pandya, 1999, 2002b), have permitted the most accurate reconstruction of the TFexcF in the human brain. We also show replication of this method in a second dataset with different scanning acquisition parameters. The TFexcF constitutes the direct monosynaptic anatomical connection utilized by the ventral network of language that enables communication between the lateral parts of the anterior to intermediate temporal region with particular prefrontal areas, including the ventrolateral language region (area 45). This communication is critical for certain aspects of processing, such as semantic language processing and selective lexical retrieval.

Future research using the anatomical criteria presented here for the reconstruction of the TFexcF will help refine our understanding of the specific role of this pathway critical for the ventral stream of language processing (Saur et al., 2008), as well as its role in working memory and selective memory retrieval (Klein et al., 1995; Petrides et al., 1995; Kostopoulos and Petrides, 2003, 2008, 2016). Accurate diffusion MRI dissections of tracts in the human brain will have a positive impact on our understanding of the networks subserving specific brain functions not only in healthy adults but also during development and aging.

## Data Availability

The data that support the findings of this study are available from the corresponding author upon reasonable request.

## Synthesis

Reviewing Editor: Ifat Levy, Yale School of Medicine Decisions are customarily a result of the Reviewing Editor and the peer reviewers coming together and discussing their recommendations until a consensus is reached. When revisions are invited, a fact-based synthesis statement explaining their decision and outlining what is needed to prepare a revision will be listed below. The following reviewer(s) agreed to reveal their identity: NONE. Note: If this manuscript was transferred from JNeurosci and a decision was made to accept the manuscript without peer review, a brief statement to this effect will instead be what is listed below.

The authors have appropriately revised in response to the j neuroscience reviews
