# Supplementary figures and images for: Dissection of the Temporofrontal Extreme Capsule Fasciculus Using Diffusion MRI Tractography and Association with Lexical Retrieval
Source: eNeuro. 2024 Jan 19;11(1):ENEURO.0363-23.2023. doi: 10.1523/ENEURO.0363-23.2023 (PMC10849018; doi:10.1523/ENEURO.0363-23.2023)

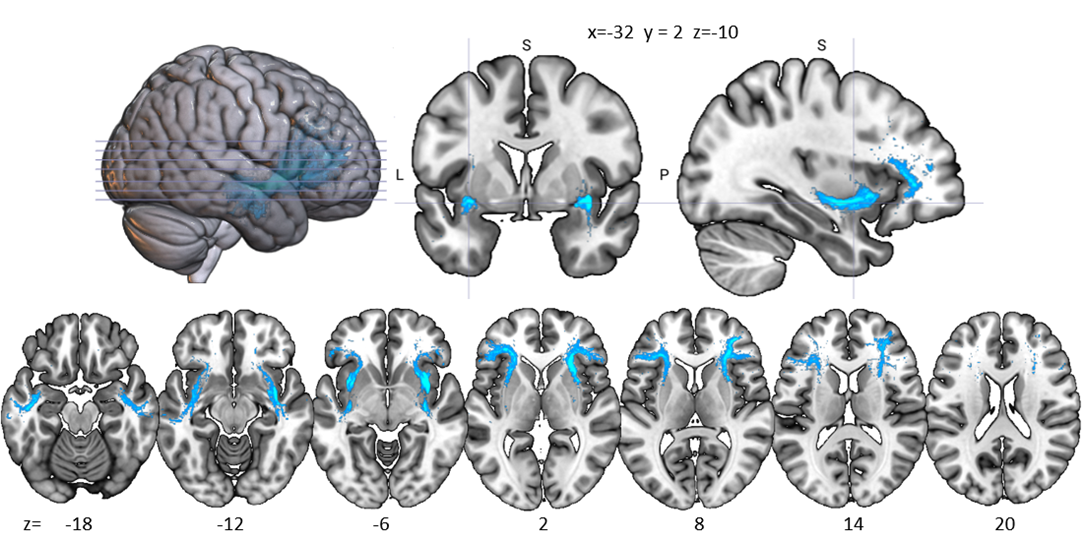

Supplement: Figure 4-1 — Left and right temporo-frontal extreme capsule fasciculus in 8 participants from the HCP dataset overlayed on the MNI brain template. Download Figure 4-1, TIF file. [file eneuro-11-ENEURO.0363-23.2023-s001.tif]
